# Genomic analysis of chromosomal cointegrated *bla*_NDM-1_-carrying ICE and *bla*_RSA-1_-carrying IME from clinical multidrug resistant *Aeromonas caviae*

**DOI:** 10.3389/fcimb.2023.1131059

**Published:** 2023-03-23

**Authors:** Xinhua Luo, Zhe Yin, Lianhua Yu, Jin Zhang, Dakang Hu, Mengqiao Xu, Peng Wang, Fengling Wang, Jiao Feng

**Affiliations:** ^1^ Department of Clinical Laboratory Medicine, Taizhou Municipal Hospital, Taizhou, China; ^2^ State Key Laboratory of Pathogen and Biosecurity, Beijing Institute of Microbiology and Epidemiology, Beijing, China; ^3^ Nanxiang Branch of Ruijin Hospital, Shanghai Jiaotong University, Shanghai, China; ^4^ Institutes of Biomedical Sciences, Shanxi University, Taiyuan, China

**Keywords:** *Aeromonas caviae*, *bla*_NDM-1_, *bla*_RSA-1_, ICE, IME

## Abstract

**Introduction:**

The objective of this study is to thoroughly analyze the detailed genomic characteristics of clinical strain 211703 of *Aeromonas caviae*, which co-carrying *bla*_RSA-1_ and *bla*_NDM-1_ genes. 211703 was isolated from the patient’s cerebrospinal fluid drainage sample in a Chinese tertiary hospital.

**Methods:**

Carbapenemase NDM was detected by the immunocolloidal gold technique. The MIC values were determined by VITEK2. The whole genome sequence of 211703 was analyzed using phylogenetics, genomic comparison, and extensive dissection.

**Results:**

This study revealed that 211703 only contained a single 4.78 Mb chromosome (61.8% GC content), and no plasmids were discovered in 211703. 15 different types of resistant genes were detected in the genome of 211703, including *bla*_RSA-1_ harbored on integrative and mobilizable element (IME) Tn*7413a*, and *bla*_NDM-1_ harbored on integrative and conjugative element (ICE). The ICE and IME were all carried on the chromosome of 211703 (c211703). Detailed comparison of related IMEs/ICEs showed that they shared similar conserved backbone regions, respectively. Comprehensive annotation revealed that *bla*_RSA-1_ was carried by the gene cassette of a novel integron In2148 on Tn*7413a*, and *bla*_NDM-1_ was captured by an insertion sequence IS*CR14*-like on the ICE of 211703. We speculated that mobile genetic elements (MGEs) such as ICE and IME facilitated the spread of resistance genes such as *bla*_RSA-1_ and *bla*_NDM-1_.

**Discussion:**

In conclusion, this study provides an overall understanding of the genomic characterization of clinically isolated *A. caviae* 211703, and an in-depth discussion of multiple acquisition methods of drug resistance genes in *Aeromonas*. To the best of our knowledge, this is the first report of *A. caviae* carrying *bla*_RSA-1_ even both *bla*_RSA-1_ and *bla*_NDM-1_, and this is the first bacterium carrying *bla*_RSA-1_ isolated from the clinical setting.

## Introduction

The genus *Aeromonas* comprises a group of Gram-negative bacterium worldwide distributed in aquatic and soil environments (Fernández-Bravo and Figueras, 2020). Among 42 different species included in the genus *Aeromonas*, three species (*Aeromonas hydrophila*, *Aeromonas caviae*, and *Aeromonas veronii*) account for the vast majority of human infections and clinical isolates (Pessoa et al., 2022). In recent years, *Aeromonas* have gradually increased, and it has become a common opportunistic pathogen that causes human infection (Bhowmick and Bhattacharjee, 2018; Pessoa et al., 2022). The main reason of carbapenem resistance in *Aeromonas* is carrying *cphA* gene encoding a CphA metallo-β-lactamase (Wu et al., 2012). In addition, *Aeromonas* has the ability to acquire different types of carbapenemase genes. In 2007, French scholars Neuwirth. C et al. reported a carbapenem-resistant clinical isolate of *A. caviae* for the first time, which produced a class B carbapenemase IMP-19 (Neuwirth et al., 2007). Subsequently, in 2008, Swedish scholars Bala´zs Libisch et al. reported the first clinical isolate of *A. hydrophila* producing VIM-4 (Libisch et al., 2008). Following this, environmental and clinical isolates of *Aeromonas* spp. producing class A carbapenemase KPC were reported in Brazil, the USA, Japan, and China, respectively (Montezzi et al., 2015; Hughes et al., 2016; Hu et al., 2019; Sekizuka et al., 2019; Yang et al., 2022). In 2017, Indian researchers Shalini Anandan and colleagues made the initial discovery of *A. caviae* strain carrying the *bla*_OXA-181_ gene, located within the composite transposon Tn*2013*. This was confirmed through whole genome sequencing. (Anandan et al., 2017). In 2018, Japanese scholar Kohei Uechi et al. reported a clinical isolate of *A. hydrophila* encoding GES-24 carbapenemase, and *bla*_GES-24_ was located in the integron (Uechi et al., 2018b). In 2022, Shuguang Xu et al. discovered an *A. caviae* strain carrying the *bla*_NDM_ gene located on its chromosome. (Xu et al., 2022). In the same year, our research team reported *bla*_NDM-1_ harboring in plasmid pK433-NDM carried in *A. caviae* K433 isolated from patient with community-acquired pneumonia (Luo et al., 2022). Overall, *Aeromonas* is becoming more prevalent in the clinic, and multidrug resistant *A. caviae* is emerging as a potential threat to human society and public health in recent years.

Ambler class B β-lactamase New Delhi metallo-β-lactamase (NDM) is one of the most common carbapenemases in *Enterobacterales* (Nordmann et al., 2011; Porreca et al., 2018; Wu et al., 2019), which can hydrolyze nearly all classes of β-lactam antibiotics. *bla*_NDM-1_, the first identified gene of 41 known variants encoding NDM, was initially detected in a *Klebsiella pneumoniae* strain in New Delhi in 2008 (Yong et al., 2009). Since then, *bla*_NDM-1_ is prevalent in various species and has rapidly spread all over the world (Dortet et al., 2014). In addition to *bla*_NDM-1_, *bla*_RSA-1_, a noval gene encoding class A β-lactamase, was first discovered and identified in Indian river sediments in 2018 (Marathe et al., 2018). Open reading frames (ORFs) of *bla*_RSA-1_ were synthesized and cloned in *E. coli* in further experiments. The clone expressing class A enzyme encoded by *bla*_RSA-1_ was verified to efficiently hydrolyze benzylpenicillin, cephalothin (first generation cephalosporin), and cefotaxime (third generation cephalosporin). The clone exhibited a β-lactam resistance phenotype typical for Extended Spectrum Beta-Lactamase (ESBL) production and showed a great resistance challenge (Marathe et al., 2018). Although *bla*_NDM-1_ and *bla*_RSA-1_ are significant drug resistance genes, strains that produce both NDM and RSA have not been reported.

Integrative and conjugative element (ICE) (Delavat et al., 2017; Botelho and Schulenburg, 2021) and integrative and mobilizable element (IME) (Bellanger et al., 2014; Guedon et al., 2017) are two types of mobile genetic elements (MGEs) primarily resided in the bacterial cell’s chromosome, and both of them have the ability to carry resistance genes. ICEs can be transferred between cells by conjugation, which function is self-encoded. The core components of an ICE typically include *attL* (attachment site at the left end of the ICE), *int* (integrase), *xis* (excisionase), *rlx* (relaxase), *nic*/*oriT* (nick site, origin of conjugative replication), *cpl* (coupling protein), a P (TivB)- or F (TivF)-type T4SS machinery (mating pair formation), and *attR* (attachment site at the right end of the ICE). IMEs are autonomous in integration but nonautonomous in conjugation. IMEs contained no conjugal transfer genes and typically have *attL*, *int*, *rlx*, *oriT*, and *attR*.

In this work, a carbapenem-resistant *A. caviae* strain 211703 co-carrying *bla*_NDM-1_ and *bla*_RSA-1_ was identified in clinical setting. According to the sequencing results, the whole genome of *A. caviae* strain 211703 contained only the chromosome (c211703), and no plasmid was found. All resistance genes including *bla*_NDM-1_ and *bla*_RSA-1_, and antimicrobial susceptibility profiles of *A. caviae* 211703 were obtained. The genetic dissection of strain 211703 of A. caviae revealed the presence of ICE carrying the *bla*_NDM-1_ gene and IME carrying the *bla*_RSA-1_ gene, both of which had integrated into the chromosome.

Comprehensive genomic comparisons of above ICE and IME with their closely related elements were performed, respectively. According to our understanding, this is the first report of *A. caviae* strain co-carrying *bla*_NDM-1_ and *bla*_RSA-1_. At the same time, this is the first report of strains carrying *bla*_RSA-1_ gene found in a clinical setting. This research will provide a new understanding for the genetic context of novel antibiotic resistance genes in *A. caviae*, and in-depth insights for the integration, transformation, conjugation, and mobilization of MGEs related to resistance genes under the selection pressure.

## Materials and methods

### Sample collection and species identification

On 10 October 2021, a patient (52-year-old male) was admitted to the neurosurgery department, due to sudden unconsciousness and vomiting for three and a half hours. The diagnosis was spontaneous subarachnoid hemorrhage, hemorrhage in the third, fourth and lateral ventricles. After admission, right ventricle drainage was performed. Fluid replacement, brain protection, prevention of bleeding infection, and neurological rehabilitation were given. On the eighth day of admission, the patient was considered intracranial infection with the body temperature 37.9°C and persistent unconsciousness, and a carbapenem-resistant *A. caviae* strain 211703 was isolated from the patient’s cerebrospinal fluid drainage sample initially identified by Vitek 2. Later, bacterial species identification using average nucleotide identity (ANI) analysis (http://www.ezbiocloud.net/tools/ani) based on genome sequences finally proved that 211703 belongs to *A. caviae* (Richter and Rossello-Mora, 2009).

### Antibiotic susceptibility test and carbapenemases phenotype detection

The drug minimum inhibitory concentrations (MICs) of 211703 were determined by BioMérieux VITEK2 (**Table 1**). The antibiotic susceptibility test results were determined by the Clinical and Laboratory Standards Institute (CLSI) guidelines (2021). The production of NDM carbapenemase in 211703 was detected on an NG-Test CARBA 5 (NG Biotech, Guipry, France), a rapid diagnostic test based on the immunocolloidal gold technique (**Figure S1**).

**Table 1:** Antimicrobial susceptibility profiles of *A. caviae* 211703.

Antibiotics	MIC values (µg/mL)	Antimicrobial susceptibility
Ceftazidime	≥64	R
Piperacilin/Tazobactam	≥128	R
Aztreonam	≤1	S
Imipenem	≥16	R
Meropenem	≥16	R
Amikacin	16	S
Ticarcillin/Clavulanic Acid	≥128	R
Ciprofloxacin	≥4	R
Levofloxacin	≥8	R
Tigecycline	≤0.5	S
Minocycline	≤1	S
Trimethoprim/Sulfamethoxazole	≥320	R

S, sensitive; R, resistant.

### Conjugal transfer

Conjugal transfer experiments were carried out with rifampicin-resistant *Escherichia coli* EC600 being used as a recipient, and the strain 211703 as a donor. The donor and recipient strains were grown in three milliliters (mL) brain heart infusion (BHI) broth overnight at 37°C. And then, 50 μL of donor strain culture was mixed with 500 μL of recipient strain culture (v:v = 1:10) and 4.5 mL of fresh BHI broth. In addition, 100 μL of the mixture was applied onto a cellulose filter membrane (pore size, 0.22 μm) already placed on a BHI agar plate. After incubation at 37°C for 16h-18h, the filter membrane was taken out and vortexed in 1 mL of BHI broth. The vortex mixtures were plated on BHI agar plates containing 2 mg/L imipenem and 1,500 mg/L rifampicin for the selection of the transconjugants. However, repeated conjugation experiments failed to acquire transconjugants.

### Sequencing and sequence assembly

Bacterial genomic DNA of *A. caviae* 211703 was isolated using the Gentra Puregene Yeast/Bact. Kit (Qiagen, Valencia, CA). The TruePrepTM DNA Library Prep Kit V2 and the SQU-LSK109 Ligation Sequencing kit were used for libraries preparation separately. After the preparation of the library was completed, it was separately sequenced on an Illumina HiSeq X Ten platform (Illumina Inc., San Diego, CA, USA) and GridION X5 platform (Oxford Nanopore, UK). Raw data from the HiSeq X Ten platform and the GridION X5 platform were trimmed to obtain the high-quality clean reads (clean data) by Canu v1.8 (https://canu.readthedocs.io/en/latest/index.html). The paired-end short Illumina reads and the long Nanopore reads were assembled *de novo* utilizing Unicycler v0.4.8.0 (https://github.com/rrwick/Unicycler).

### Whole-genome phylogeny and genetic background analysis

**Figure 1:**
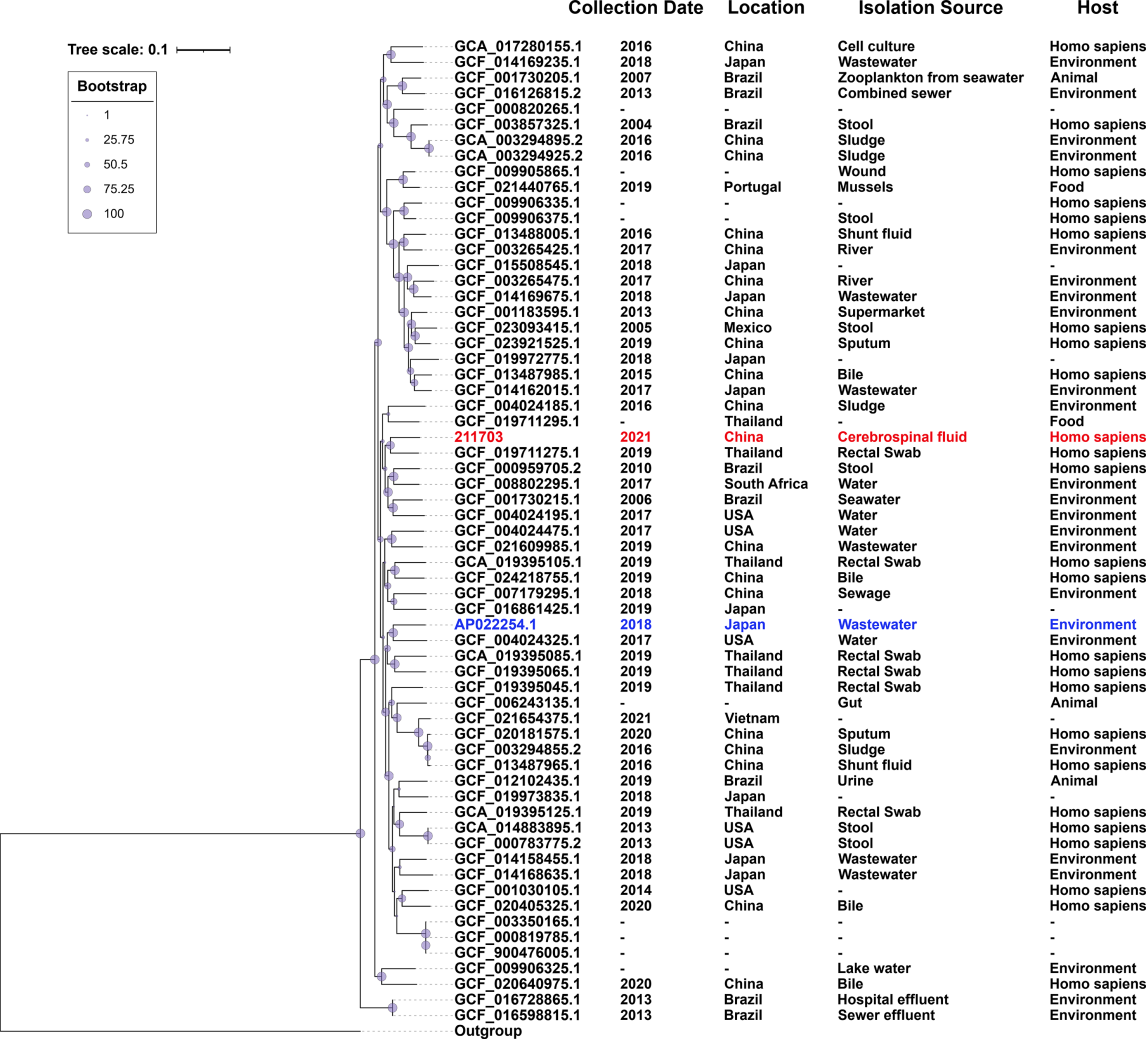
Population distribution of *A. caviae* 211703 with 62 A. *caviae* genomes. The phylogenetic tree was constructed by the Maximum-likelihood method. The degree of support (percentage) for each cluster of associated taxa, as determined by bootstrap analysis, was shown with blue dots next to each branch. The bar corresponded to the scale of sequence divergence. 211703 was indicated in red and AP022254.1 was indicated in blue.

A total of 62 sequences of *A. caviae* sequenced at three levels (scaffold, chromosome, and complete) were downloaded from NCBI (last accessed on 15 Aug 2022), which were isolated from various sources from 2004 to 2021 (**Table S1**). Assembly method of 211703 genome that were used in the phylogenetic analysis was Unicycler v0.4.8.0. MUMmer v3.1 was used for alignments against the reference genome to create a core genome alignment (Delcher et al., 2003). The core-genome length for phylogenetic analysis was 4.5-Mb. A total of 394,778 single nucleotide polymorphisms (SNPs) in the backbone regions were identified and extracted, and a maximum-likelihood phylogenetic tree was constructed based on the SNPs’ dataset (**Figure 1**). Matrix of pairwise SNP distances among studied genomes was shown in **Table S2**. The phylogenetic tree and related background information (collection date, location, isolation source, and host) were shown using the Interactive Tree of Life (iTOL) programs (Letunic and Bork, 2021).

### Sequence annotation and comparison

ORFs and pseudogenes of 211703 genome were predicted using RAST 2.0 (Brettin et al., 2015). Further detailed dissection and manual annotation were performed using BLASTP/BLASTN (Boratyn et al., 2013). Annotation of resistance genes, MGEs, and other features was carried out using online databases such as CARD (Alcock et al., 2020), ResFinder (Zankari et al., 2012), ISfinder (Siguier et al., 2006), and INTEGRALL (Moura et al., 2009), and the Tn Number Registry (Roberts et al., 2008). Alignments with homologous sequences of ICE harboring on 211703 were performed by using the BRIG tool (Alikhan et al., 2011). Multiple and pairwise sequence comparisons were performed using MUSCLE 3.8.31 and BLASTN (Edgar, 2004). Comparison diagrams of ICEs/IMEs regions were drawn using Inkscape 1.1 (https://inkscape.org/en).

### Nucleotide sequence accession number

The complete sequence of *A. caviae* 211703 (chromosome c211703) was submitted to the GenBank database, under accession number CP092181. The Illumina reads of *A. caviae* 211703 was deposited as a SRA in the GenBank database (accession number PRJNA934671).

## Results

### Antimicrobial susceptibility test

Antimicrobial susceptibility tests were performed on *A. caviae* 211703. The results showed that 211703 exhibited resistance to a broad range of tested antimicrobials except aztreonam, amikacin, tigecycline, and minocycline (**Table 1**). The production of NDM carbapenemase was confirmed by immunocolloidal gold technique, which was consistent with the result of antibiotic susceptibility tests (**Figure S1**). Corresponding to the results of multidrug resistance profile, 15 different types of resistant genes were detected from *A. caviae* 211703 (**Table S3**), which confer resistance to aminoglycosides (*arr2* and *aphA6*), β-lactams (*bla*_MOX-6_, *bla*_NDM-1_, and *bla*_RSA-1_), macrolides (*mph*(A), *mph*(E), and *msr*(E)), quinolones (*qnrVC1*), sulphonamides (*sul1*), and other antibiotics.

### Phylogenetic Analysis of *Aeromonas caviae* 211703

Based on the genome of 211703 and all *A. caviae* strains available from GenBank of NCBI whose assembly levels are scaffold, chromosome, or complete, phylogenetic analysis was performed to explore the evolutionary relationships and potential associations (Sayers et al., 2021). A total of 62 A*. caviae* strains from GenBank were included, and the related information of these strains were collected and verified manually (**Table S1**). From the background information, the main collection sites of these strains were China (18/62), Japan (10/62), Brazil (9/62), Thailand (7/62), and the USA (6/62). Among these 62 strains, the chromosome sequence of *A. caviae* WP8-S18-ESBL-04 (accession number AP022254, GCF_014169735.1, the standard strain of *A. caviae*) was used as the reference. Strain OnP3.1 (accession number CP050851, GCF_017310215.1, the standard strain of *A. hydrophila*) was used as the outgroup. After multiple sequence alignment, a total of 394,778 SNPs located in the core genome regions were identified and extracted. Using these SNPs’ dataset, a maximum likelihood (ML) phylogenetic tree was constructed. **Figure 1** showed the phylogenetic tree and marked basic background information of each node/strain such as the collection date, location, isolation source, and host. The most closely related strain to 211703 was GCF_019711275.1, which was isolated from a rectal swab in Thailand in 2019. They both belonged to the same branch on the evolutionary tree.

### Overview of the *Aeromonas caviae* 211703

The whole genome sequence of *A. caviae* 211703 was obtained by high-throughput sequencing. Genome sequencing result revealed that *A. caviae* 211703 contained only chromosome (c211703, accession number CP092181) with a length of 4,783,384 bp, and no plasmid was found in its genome after verification (**Table 2**). The mean G+C content of c211703 was 61.8% and 4,400 ORFs were predicted on c211703. Based on the six key housekeeping genes (*gltA*, *groL*, *gyrB*, *metG*, *ppsA*, and *recA*) identified on c211703, MLST typing method proved that 211703 belongs to ST928.

**Table 2:** Whole genome information of *A. caviae* 211703.

Sequence	Mean G+C content (%)	Length (bp)	Total number of ORFs	MLST	Accession number
c211703	61.8%	4,783,384	4,400	ST928	CP092181

Among the 16 resistance locus existing on c211703, 15 of them were concentrated on one ICE (6/16, including *bla*_NDM-1_) and one IME (9/16, including *bla*_RSA-1_) (**Table S3**; **Figure S2**). Further, detailed genome annotation identified the location and genetic context of ICE and IME (designated as Tn*7413a*). In order to explore the similarities and differences in the acquisition of resistance genes and the genetic environment, precise comparisons were performed with related ICEs and IMEs, respectively.

### Genetic characterization of the IME Tn*7413a*

**Figure 2:**
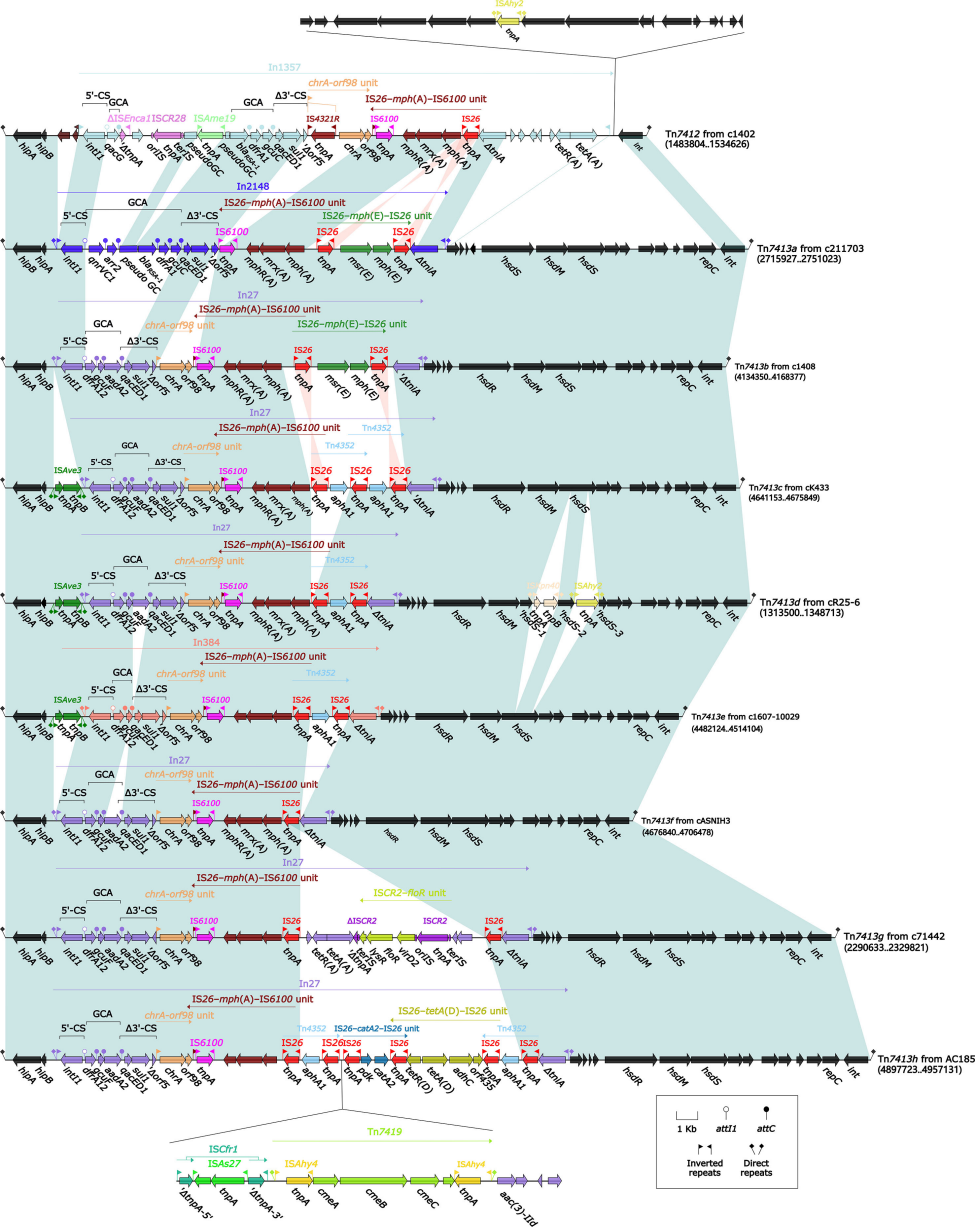
Comparison of Tn*7413a* with related IMEs. Genes were denoted by arrows. Genes, mobile genetic elements, and other features were colored based on their functional classification. Shading denoted regions of homology (nucleotide identity > 95%). A single quotation mark in front of the gene name indicated pseudogene.

The IME Tn*7413a* carrying *bla*_RSA-1_ was 35,097 bp in length, located on *A. caviae* 211703 from 2,715,927 bp to 2,751,023 bp (**Figure S2**). Accurate annotation and genomic dissection revealed that Tn*7413a* was bordered by a pair of 18-bp *attL*/*attR* (attachment site at the left/right end) (**Figure 2**). According to genes function, Tn*7413a* can be divided into backbone regions and accessory regions. Backbone regions with a length of 16.2 kb were consisted by genes such as *int*, *repC*, *hipA*, and *hipB*. Integron In2148 inserted into the backbone regions, as the accessory region of Tn*7413a*.

In2148 was a novel concise class 1 integron containing primary components 5’-conserved region (5’-CS), 3’-conserved region (3’-CS), and a gene cassette array (GCA) *qnrVC1*–*arr2*–*bla*_RSA-1_–*dfrA1*–*gcuC*. The *bla*_RSA-1_ gene was found to be located in the GCA of In2148, most likely due to the integration of an integron, which facilitated its spread. In the downstream of 3’-CS, there was a reverse putative resistance unit IS*26*–*mph*(A)–IS*6100* unit carrying *mph*(A), encoding proteins conferring resistance to macrolides (Partridge et al., 2018). In the upstream of IS*26*–*mph*(A)–IS*6100* unit, IS*26* was shared by IS*26*–*mph*(E)–IS*26* unit followed by a truncated *tniA* encoding DD(35)E transposase TniA. A pair of 25-bp IRi (inverted repeat at the integrase end) and IRt (inverted repeat at the *tni* end) flanked In2148. On the outside of IRi and IRt, there were 5-bp DRs (direct repeats, target site duplication signals for transposition), respectively.

### Eight Tn*7413a*-related IMEs Tn*7413b-h* and Tn*7412*

To investigate the structural variations among these IMEs, a BLAST analysis was performed using the backbone region sequences of Tn*7413a* as a reference. BLAST analysis revealed that a total of seven IMEs in the GenBank database were similar to Tn*7413a*, sharing 100% coverage and >99% identity for gene *int* from Tn*7413a*. These seven IMEs were included in this analysis, and the gene structures of these IMEs were detailed annotated manually. Due to their structural similarity to Tn*7413a*, these seven IMEs were designated as Tn*7413b-h* as listed in **Table S4**.

Tn*7413b-h* ranged in length from 29,639 bp to 59,408 bp, and were all distributed in *Aeromonas*. Similar to Tn*7413a*, the boundaries of these seven IMEs were a pair of 18-bp *attL*/*attR*. The backbone regions of Tn*7413b-h* were almost identical to that of Tn*7413a*, but differed in the accessory regions (**Figure 2**). In all the seven IMEs except Tn*7413d*, there was only one accessory region with integron as the primary content. Two insertion sequences insertions in Tn*7413d* resulted in its backbone gene *hsdS* being interrupted into three parts. In Tn*7413b*-*d* and Tn*7413f*-*h*, In2148 in Tn*7413a* was replaced by In27. In27 was a concise class 1 integron, carrying GCA *dfrA12*–*gcuF*–*aadA2* (Partridge et al., 2009; Partridge, 2011). In Tn*7413e*, In2148 in Tn*7413a* was replaced by In384, a concise class 1 integron carrying GCA *dfrA12*–*gcuF*. In Tn*7413c-e*, the insertion sequence IS*Ave3* inserted upstream of the integron. For all these seven IMEs Tn*7413b-h*, there was the putative resistance unit *chrA–orf98* unit downstream of the 3’-CS of the integron they carried. Further, the *chrA–orf98* unit was all followed by an IS*26*–*mph*(A)–IS*6100* unit, which was same as in Tn*7413a*. For Tn*7413c-e* and Tn*7413h*, all contained single or multiple Tn*4352*, a composite transposon involved in kanamycin resistance (Wrighton and Strike, 1987). For Tn*7413b*, the most identical IME related to Tn*7413a*, its integron In27 contained the IS*26*–*mph*(E)–IS*26* unit, which was identical to Tn*7413a*. Tn*7413g* contained a putative resistance unit IS*CR2*–*floR* unit. IS*CR* elements were bounded by an origin (*oriIS*) downstream and a terminus (*terIS*) upstream, responsible for capturing resistance genes (Partridge, 2011; Partridge et al., 2018). In Tn*7413h*, there was a novel composite transposon newly designated Tn*7419*, flanked by 8-bp DRs both sides. Both ends of Tn*7419* were insertion sequence IS*Ahy4*, and the middle part were *cmeA*, *cmeB*, and *cmeC*, encoding membrane fusion proteins related to RND efflux system. Meanwhile, downstream of Tn*7419*, there were IS*26*–*catA2*–IS*26* unit and IS*26*–*tetA*(D)–IS*26* unit sharing IS*26*, carrying resistance genes *catA2* and *tetA*(D), respectively.

Furthermore, in order to explore the genetic environment differences of *bla*_RSA-1_, the genetic environment of the previously reported *bla*_RSA-1_, which belonged to *Aeromonas* spp. strain 1402 was also included in this study (Marathe et al., 2018). The results showed that the gene fragment carrying *bla*_RSA-1_ in former report was an IME with a length of 50,823 bp, which was also bounded by a pair of 18-bp length *attL*/*attR* (**Figure 2**). Backbone regions of Tn*7412* also contained genes such as *int*, *hipA*, and *hipB*, but other genes in the backbone region were quite different from Tn*7413a* and Tn*7413b-h*. The accessory region of Tn*7412* was mainly composed of concise class 1 integron In1357, containing GCA *qacG*–*bla*_RSA-1_–*dfrA1*–*gcuC*. Coincidentally, *bla*_RSA-1_ was also located in the gene cassette of the integron, consistent with Tn*7413a*. After the 3’-CS of In1357, there were *chrA–orf98* unit and IS*26*–*mph*(A)–IS*6100* unit.

### Genetic characterization of ICE of *Aeromonas caviae* 211703

**Figure 3:**
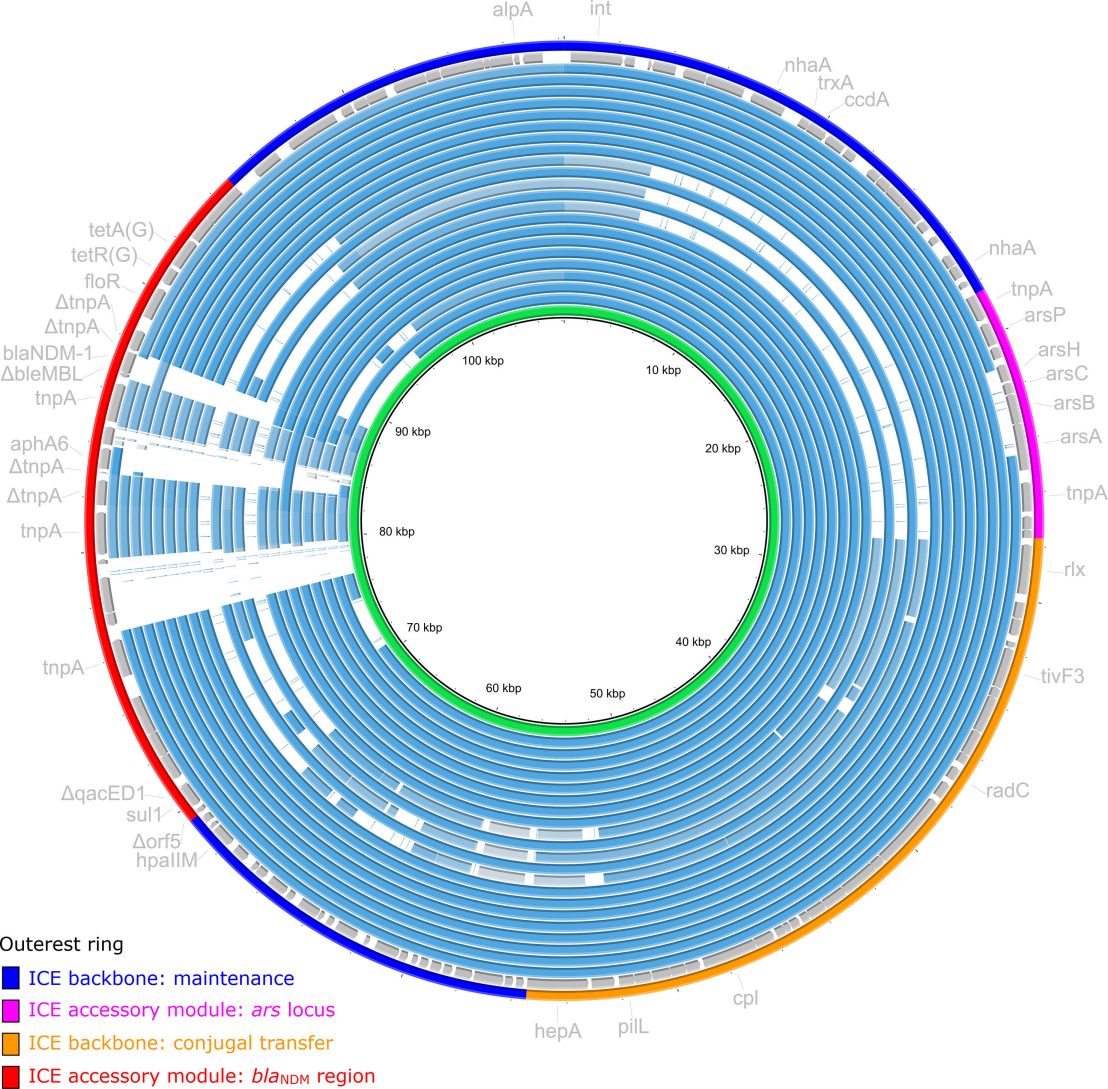
Alignment of ICE of *A. caviae* 211703 with related ICEs. ICE of *A. caviae* 211703 with 21 other similar ICEs deposited in the GenBank database were included. The rings of plasmids were arranged in the order (from inner to outer) as described in **Table S5**. The second outer ring colored in gray were annotations of ICE of *A. caviae* 211703. Highlighted on the outer ring represented functional regions (backbone regions and accessory regions).

**Figure 4:**
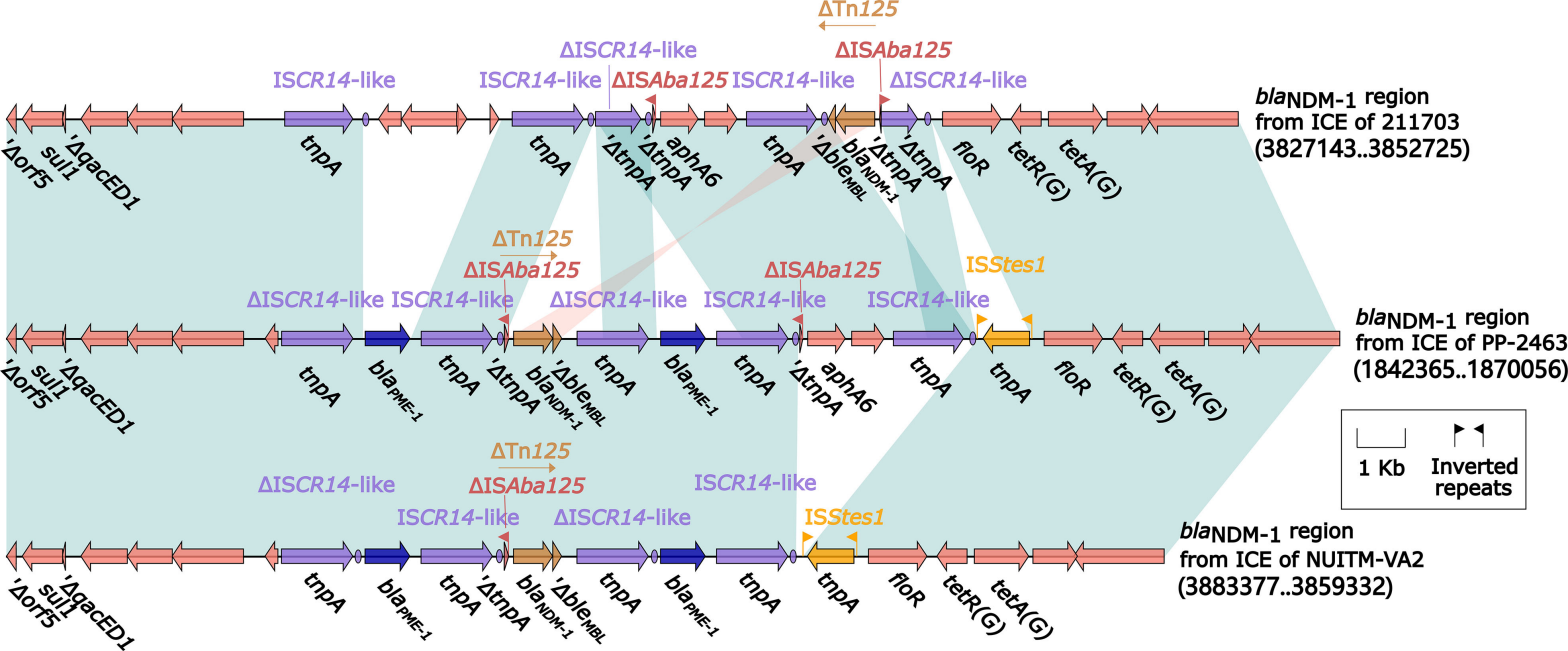
Comparison of ICEs harboring *bla*_NDM-1_ region from 211703, PP-2463, and NUITM-VA2. Genes were denoted by arrows. Genes, mobile genetic elements, and other features were colored based on their functional classification. Shading denoted regions of homology (nucleotide identity > 95%). A single quotation mark in front of the gene name indicated pseudogene.

ICE of *A. caviae* 211703 was located on chromosome from 3,757,651 bp to 3,865,845 bp with a length of 108,195 bp, bounded by a pair of 19-bp *attL*/*attR* (**Figure S2**). Genomic annotation revealed that ICE of *A. caviae* 211703 can also be divided into backbone regions and accessory regions according to their functions (**Figure 3**). The backbone regions consisted of two parts, including backbone of maintenance harboring gene *int* and backbone of conjugal transfer. Accurate annotation demonstrated that there were two accessory regions on *A. caviae* 211703, *ars locus* and *bla*_NDM-1_ region. *ars locus* were a series of resistance genes encoding proteins related to arsenic resistance. *bla*_NDM-1_ region from ICE of 211703 was formed by multiple recombination mediated by IS*CR14*-like (**Figure 4**). Unlike common structures of genetic contexts harboring *bla*_NDM-1_ previously reported (ΔTn*125* harboring *bla*_NDM-1_ captured by IS*CR1*) (Wu et al., 2019; Luo et al., 2021), the *bla*_NDM-1_ genetic context was formed by the insertion sequence IS*CR14*-like mediated capture of ΔTn*125* harboring *bla*_NDM-1_ and *ble*_MBL_. Downstream of ΔIS*Aba125* were another IS*CR14*-like with *floR*, *tetR*(G), and *tetA*(G), which together constituted the end of the *bla*_NDM-1_ region from ICE of 211703. There were four IS*CR14*-like elements upstream of the IS*CR14*-like captured ΔTn*125*, which indicated that multiple insertions or recombination occurred.

BLAST analysis revealed that a total of 21 ICEs in the GenBank database were almost identical to ICE of *A. caviae* 211703 (100% coverage and >95% identity for gene *int* from ICE of *A. caviae* 211703) (**Table S5**). The 21 ICEs ranged in length from 88,228 bp to 112,147 bp. Most of these ICEs (14/21) were distributed in *Pseudomonas*, where ICE of *Pseudomonas juntendi* PP-2463 was highly identical to ICE of *A. caviae* 211703. P*. juntendi* PP-2463 also isolated from the same city (Taizhou, China) with *A. caviae* 211703, which indicated that ICE of *A. caviae* 211703 may be transferred from *Pseudomonas* to *Aeromonas*. Only one of the 21 ICEs originated from *Aeromonas* (ICE of NUITM-VA2). As shown in **Figure 3**, most of these ICEs (17/21) maintained high concordance with the ICE of 211703 in the backbone region of maintenance, *ars locus*, and the backbone region of conjugal transfer, and the differences mainly occurred in the accessory region where the *bla*_NDM-1_ region was located.

Among the 21 ICEs, the ICE of *P. juntendi* PP_2463 (sharing the most similar accessory regions with the ICE of *A. caviae* 211703) and the ICE of *A. caviae* NUITM-VA2 (the only one ICE from *A. caviae*), were specially annotated and analyzed. Detailed annotation of their *bla*_NDM-1_ region and comparison with *bla*_NDM-1_ region from ICE of 211703 were performed (**Figure 4**). Results showed that the genetic contexts of *bla*_NDM-1_ region from ICE of PP-2463/NUITM-VA2 shared a common structure in upstream(Δ*orf5*–*sul1*–Δ*qacED1*–IS*CR14*-like) with *bla*_NDM-1_ region from ICE of 211703, following by a replacement of gene *bla*_PME-1_. The genetic structures identified included an IS*CR14*-like ΔTn*125* carrying the *bla*_NDM-1_ gene, as well as a ΔIS*CR14*-like–*bla*_PME-1_–IS*CR14*-like structure, which was not present in the ICE carrying *bla*_NDM-1_ in strain 211703. The only difference found between the *bla*_NDM-1_ regions from ICE of PP-2463 and NUITM-VA2 was an insertion of *aphA6*–IS*CR14*-like, which was also present in the *bla*_NDM-1_ region from ICE of 211703. Despite this variation, all three *bla*_NDM-1_ regions shared a common structure downstream, consisting of *floR*–*tetR*(G)–*tetA*(G). However, there was an additional insertion of the IS*Stes1* sequence downstream of the *bla*_NDM-1_ regions in both ICE of PP-2463 and NUITM-VA2.

## Discussion

Carbapenem-resistant *Aeromonas* has received more attention since the first isolate from France harboring an acquired *bla*_IMP_ gene was reported in 2007 (Neuwirth et al., 2007; Pessoa et al., 2022). So far, carbapenem resistance genes such as *bla*_KPC_ (Montezzi et al., 2015), *bla*_NDM_ (Luo et al., 2022), *bla*_OXA_ (Anandan et al., 2017), and *bla*_GES_ (Uechi et al., 2018a) have been reported many times in *Aeromonas*. From a geographic perspective, carbapenem-resistant *Aeromonas* were distributed almost all over the world, such as the USA (Hughes et al., 2016), China (Luo et al., 2022), Japan (Sekizuka et al., 2019), Brazil (Conte et al., 2022), and Europe (Neuwirth et al., 2007). From the perspective of host distribution, *Aeromonas* were reported in different hosts such as human (Luo et al., 2022) and environment (river sediment, wastewater treatment plant effluent, and hospital effluent) (Hu et al., 2019; Sekizuka et al., 2019; Conte et al., 2022). In the “One Health” approach, the emergence of *Aeromonas* carrying antibiotic resistance genes indicates that the drug-resistant *Aeromonas* is becoming an important threat to human public health. To the best of our knowledge, the newly discovered strain 211703 of A. caviae in this study, which carrying the novel *bla*_RSA-1_ gene, is the first such bacterium to be isolated from a clinical setting worldwide. It is also the first report of *Aeromonas* harboring a new drug resistance gene combination of *bla*_RSA-1_ and *bla*_NDM-1_. At the same time, the *bla*_RSA-1_ and *bla*_NDM-1_ gene in this study were acquired by *A. caviae* in two different ways, indicating that the drug resistance of *A. caviae* changes rapidly under selection pressure, marking a further increase in the threat of *A. caviae* to human society.

The *bla*_RSA-1_ gene was first discovered in Indian river sediments in 2018 (Marathe et al., 2018). Through the verification of metagenomics and drug susceptibility experiments, as well as phylogenetic analysis, the *bla*_RSA-1_ gene was determined to be a new beta-lactam antibiotic resistance gene. In previous study, the *bla*_RSA-1_ gene was located in the integron gene cassette, but the gene environment was not analyzed by detailed annotation. After 2018, there has been no related report of *bla*_RSA-1_ gene. In this study, we identified strain *A. caviae* 211703 from clinical setting carrying the *bla*_RSA-1_ gene. Compared with previous report, we speculated the *bla*_RSA-1_ gene was transferred from India to China geographically, and from the river sediments to the clinic in terms of infection environment. The only similarity is that both of the *bla*_RSA-1_ genes are located in the gene cassette of the integron. We carried out detailed annotation and comparative genomics analysis of the gene environment where the *bla*_RSA-1_ is located, and found that there are many IMEs (Tn*7413b-h*) that are similar in structure to the IME (Tn*7413a*) where the integron In2148 carrying *bla*_RSA-1_ is located (**Figure 2**; **Table S4**). The IMEs Tn*7413a-h* backbone regions are relatively conserved and intact, although their accessory regions have undergone recombination events such as replacement or insertion. The IMEs Tn*7413a-h* are all derived from *Aeromonas* and have highly conserved and homologous backbone regions, indicating a widespread distribution of this category of IMEs within *Aeromonas*. At the same time, the *bla*_RSA-1_ gene is located in the gene cassette of In2148, which can be speculated that the gene *bla*_RSA-1_ carrying by the IME Tn*7413a* was derived by the capture from integron. Therefore, it is foreseeable that the *bla*_RSA-1_ gene cassette has gained a stronger capability to spread. In addition, the IME Tn*7413a* harboring *bla*_RSA-1_ is likely to spread in *Aeromonas* through conjugal transfer and other methods, although the IME itself does not have the ability to transfer. Its threat cannot be underestimated.

*bla*_NDM-1_ is a drug resistance gene encoding carbapenemase, which has strong resistance to carbapenems, and has been widely reported in *Enterobacterales* all over the world (Nordmann et al., 2011; Porreca et al., 2018; Wu et al., 2019). In *Aeromonas*, *bla*_NDM-1_ was discovered in the last year or two (Wang et al., 2021; Luo et al., 2022). In the strain *A. caviae* 211703 of this study, the genetic environment in which *bla*_NDM-1_ is located is slightly different from the previous reports (**Figure 4**). The ΔTn*125* containing *bla*_NDM-1_ in the strain *A. caviae* 211703 was carried by the ICE of the 211703 chromosome (c211703), and was captured by IS*CR14*-like rather than the common IS*CR1*, and was not further integrated into the integron. In addition, more than one insertion and recombination of IS*CR14*-like related elements occurred in the *bla*_NDM-1_ region from ICE of 211703, and similar phenomenon also appeared in the *bla*_NDM-1_ regions of *Pseudomonas* PP-2463 and *Aeromonas* NUITM-VA2. The *bla*_NDM-1_ regions from 211703, PP-2463, and NUITM-VA2 showed high homology, indicating that such *bla*_NDM-1_ environments have existed across species. This new *bla*_NDM-1_ genetic environment may herald a new way of recombination, which needs to be focused on. Further, the similar related ICE included in this study are mainly derived from *Pseudomonas* (**Figure 3**; **Table S5**). The ICE with the highest homology to ICE in *A. caviae* 211703 was also isolated in *Pseudomona* (PP-2463), and a high degree of structural similarity was shared by *bla*_NDM-1_ regions from both *A. caviae* 211703 and *Pseudomonas* PP-2463. This may indicate that the ICE in *A. caviae* 211703 was transferred from *Pseudomonas*.

## Conclusion

In this work, we characterized and deciphered the genomic features and population distribution of 211703, a newly identified extensively drug resistant *A. caviae* strain harboring both *bla*_RSA-1_ and *bla*_NDM-1_ isolated from the clinical patient. To the best of our knowledge, this is the first report of *A. caviae* harboring *bla*_RSA-1_ even both *bla*_RSA-1_ and *bla*_NDM-1_ from clinical setting in the world. For the first time, we detailed annotated the integron where the *bla*_RSA-1_ gene is located and deeply analyzed its related genetic environment. Further, the uncommon genetic environment of *bla*_NDM-1_ was discovered in this study and comparative genomics analysis was performed. The coexistence of *bla*_RSA-1_ and *bla*_NDM-1_ can result in more extensive antibiotic resistance in the host bacteria, and is a significant threat to public health. This study would not only provide an overall understanding of the genomic characterization of clinically isolated carbapenem-resistant *A. caviae*, but also provide an in-depth discussion of the multiple acquisition methods of drug resistance genes in *Aeromonas*. We hope that this study will contribute to the understanding of the drug resistance of *Aeromonas*, and provide a richer perspective on how drug resistance genes are carried in *Aeromonas*.

## Data availability statement

The datasets presented in this study can be found in online repositories. The names of the repository/repositories and accession number(s) can be found in the article/**Supplementary Material**.

## Ethics statement

The specimens were obtained with the patient’s consent. The use of human specimens and all related experimental protocols were reviewed and approved by the Ethics Committee of Taizhou Municipal Hospital, Zhejiang, China, in accordance with the medical research regulations of the Ministry of Health, China. Research and all related procedures involving biohazardous materials were approved by the Biosafety Committee of Taizhou Municipal Hospital. This research was conducted in China.

## Author contributions

Conceptualization, XL, JF, ZY; and FW; methodology, XL, JZ and MX; data analysis, JF, XL, JZ, LY; resources, LY, DH, and PW; writing—original draft, XL and ZY; writing—review and editing, FW and JF. All authors contributed to the article and approved the submitted version.

## Supplementary material

Supplementary Figure 1 Confirmation of production of NDM.

Supplementary Figure 2 Location of ICE and IME on *A. caviae* 211703.

Supplementary Table 1 Background of isolates included in phylogenetic analysis.

Supplementary Table 2 Pairwise.

Supplementary Table 3 Resistance genes in *A. caviae* 211703.

Supplementary Table 4 Major features of IMEs characterized in this work.

Supplementary Table 5 Major features of ICEs characterized in this work.
