# Supplementary figures and images for: Genomic analysis of chromosomal cointegrated bla NDM-1-carrying ICE and bla RSA-1-carrying IME from clinical multidrug resistant Aeromonas caviae
Source: Front Cell Infect Microbiol. 2023 Mar 23;13:1131059. doi: 10.3389/fcimb.2023.1131059 (PMC10076717; doi:10.3389/fcimb.2023.1131059)

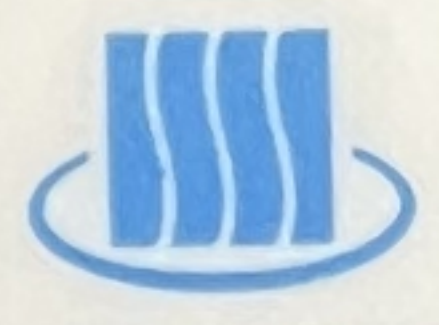 金山川  
GOLD MOUNTAIN RIVER

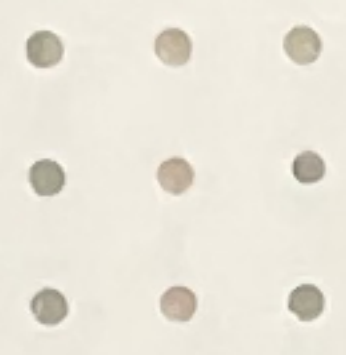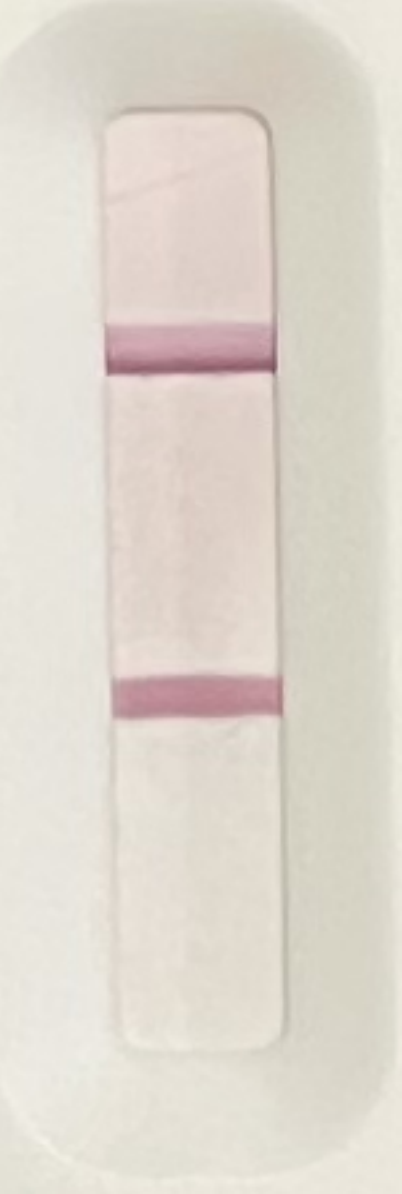

C  
T

ID: \_\_\_\_\_

S

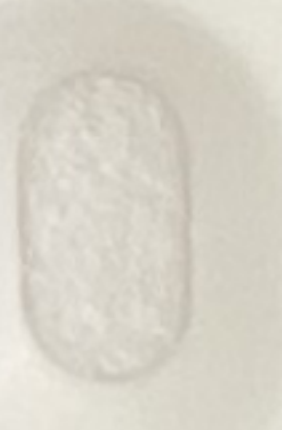

**NDM**

Supplement: Supplementary Figure 1 — Confirmation of production of NDM. [file DataSheet_1.pdf]

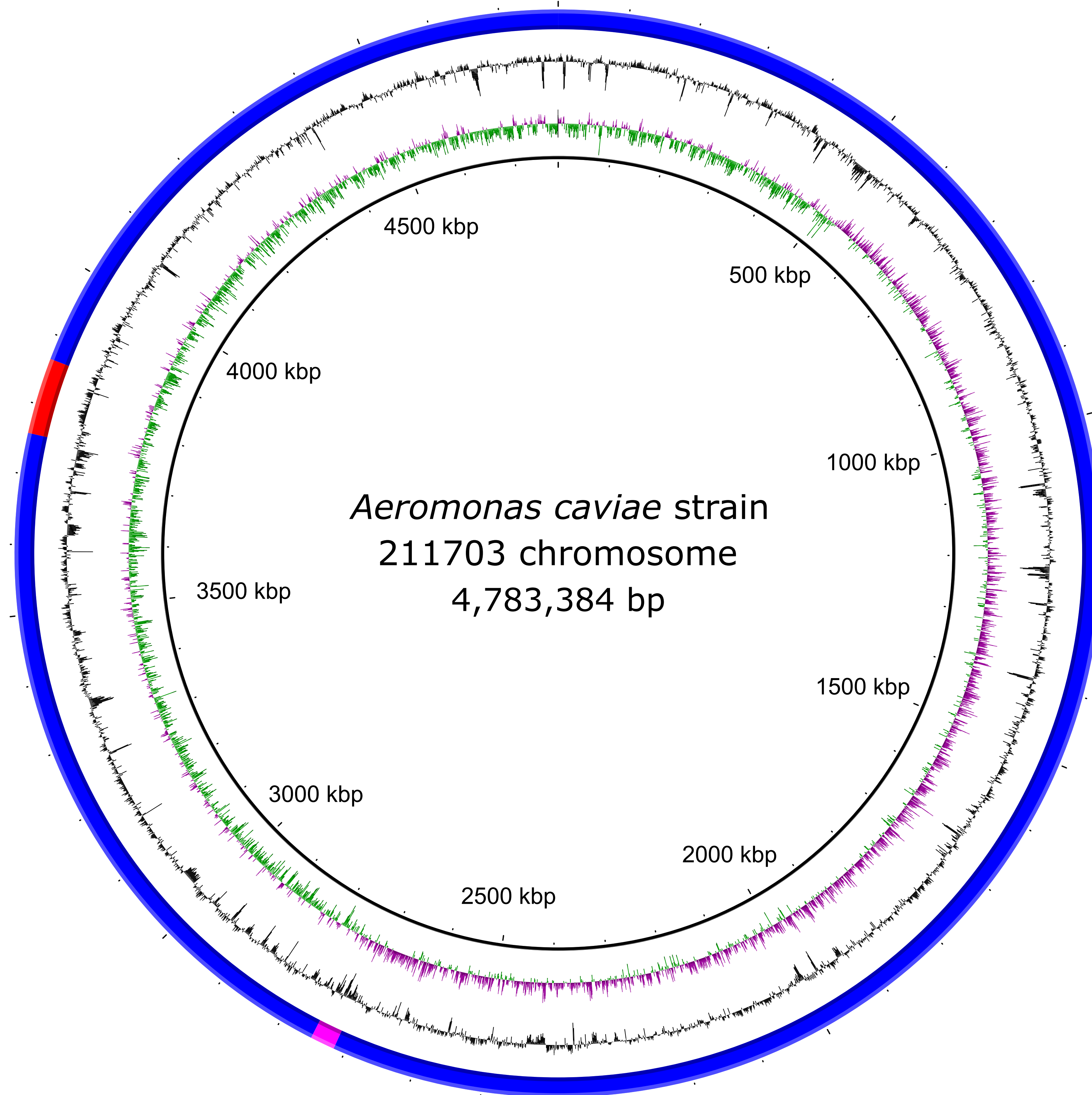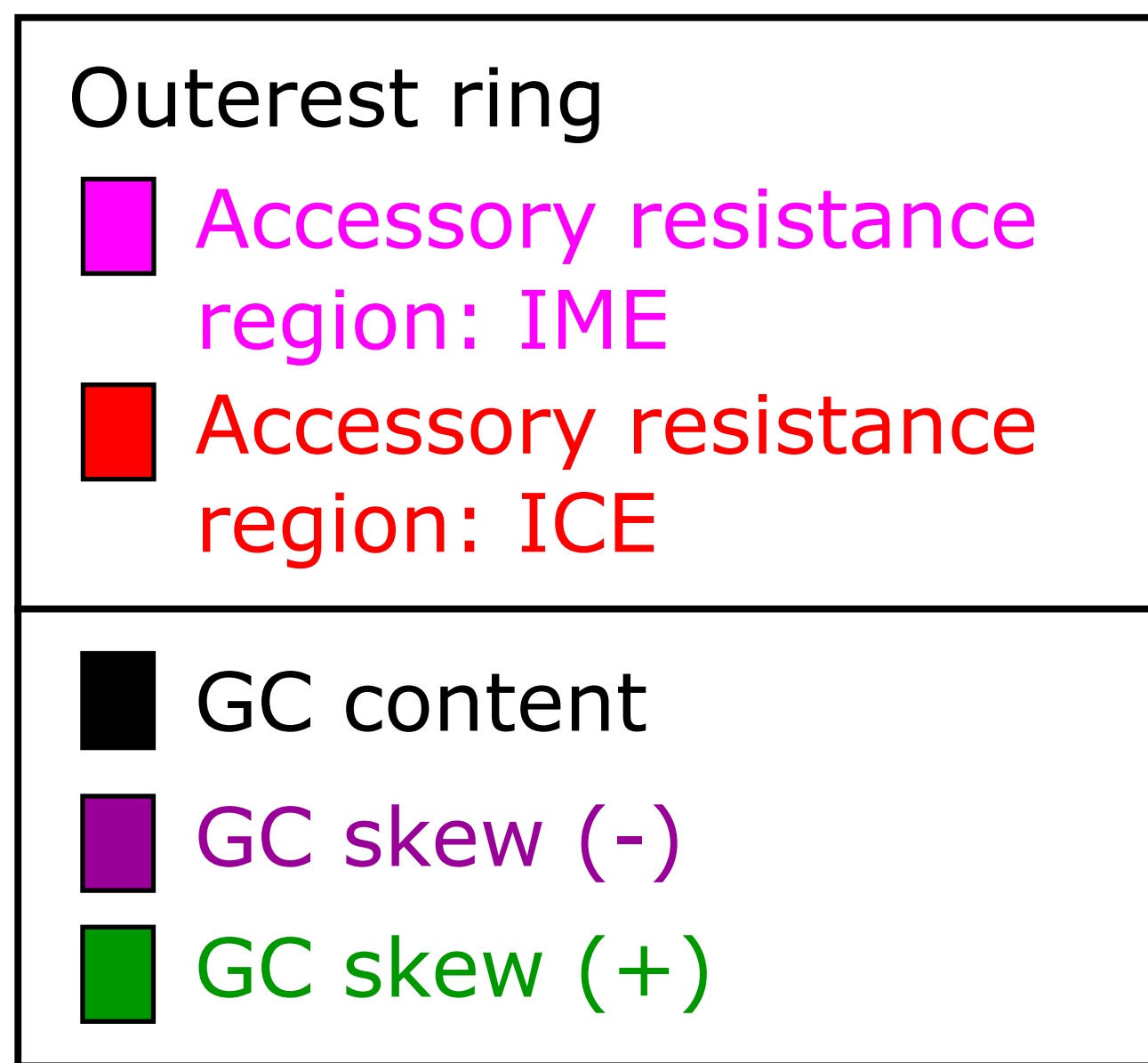

Supplement: Supplementary Figure 2 — Location of ICE and IME on A. caviae 211703. [file DataSheet_2.pdf]
