# Supplementary material for: Genomic analysis of chromosomal cointegrated bla NDM-1-carrying ICE and bla RSA-1-carrying IME from clinical multidrug resistant Aeromonas caviae
Source: Front Cell Infect Microbiol. 2023 Mar 23;13:1131059. doi: 10.3389/fcimb.2023.1131059 (PMC10076717; doi:10.3389/fcimb.2023.1131059)
Supplement: Supplementary Table 5 — Major features of ICEs characterized in this work. [file Table_5.docx]

**Table S2|Resistance genes in *A. caviae* 211703**

| **Sequence** | **Resistance locus** | **Resistance phenotype** | **Nucleotide position** | **Region located** |
| --- | --- | --- | --- | --- |
| c211703 | *mph*(E) | Macrolide resistance | 2732771..2733655 | Tn*7413a* |
|  | *msr*(E) | Macrolide resistance | 2733711..2735186 |  |
|  | *mph*(A) | Macrolide resistance | 2736911..2737816 |  |
|  | *sul1* | Sulphonamide resistance | 2741408..2742247 |  |
|  | *qacED1* | Quaternary ammonium resistance | 2742241..2742588 |  |
|  | *dfrA1* | Trimethoprim resistance | 2743292..2743765 |  |
|  | *bla*_RSA-1_ | β-lactam resistance | 2743892..2744749 |  |
|  | *arr2* | Aminoglycoside resistance | 2745755..2746207 |  |
|  | *qnrVC1* | Quinolone resistance | 2746383..2747078 |  |
|  | *ars* locus | Arsenic resistance | 3777151..3785336 | ICE |
|  | *sul1* | Sulphonamide resistance | 3827470..3828309 |  |
|  | *aphA6* | Aminoglycoside resistance | 3840729..3841508 |  |
|  | *bla*_NDM-1_ | β-lactam resistance | 3844363..3845175 |  |
|  | *floR* | Phenicol resistance | 3846585..3847799 |  |
|  | *tetA*(G) | Tetracycline resistance | 3848784..3849911 |  |
|  | *bla*_MOX-6_ | β-lactam resistance | 4183960..4185111 | c211703 backbone |
